# cGAS-STING-mediated inflammation and neurodegeneration as a strategy for the treatment of neurodegenerative diseases: Role of mtDNA-cGAS-STING in neurodegenerative diseases

**DOI:** 10.3724/abbs.2023258

**Published:** 2023-12-07

**Authors:** Yongjia Luo, Zhenwang Zhao, Xiaobo Hu

**Affiliations:** 1 Department of Biochemistry and Molecular Biology School of Basic Medicine Hengyang Medical School University of South China Hengyang 421001 China; 2 Department of Pathology and Pathophysiology School of Basic Medicine Health Science Center Hubei University of Arts and Science Xiangyang 441053 China

Aging poses a significant global health challenge due to its association with various diseases, including cancers, cardiovascular diseases, diabetes, and particularly neurodegenerative diseases. Extensive evidence has suggested cellular senescence plays a critical role in the initiation and progression of ageing-associated diseases. Under pathological conditions, senescent cells exhibit intrinsic disorders and secrete various bioactive molecules, referred to as senescence-associated secretory phenotype (SASP), which results in chronic inflammation and impaired tissue function. Aging is commonly accompanied with systemic low-grade inflammation (named inflammaging), contributing to organ damage and ageing-related impairment and diseases. Hence, alleviating inflammaging could serve as a potential strategy for anti-aging and anti-ageing-related diseases.

The cyclic GMP-AMP synthase (cGAS)-stimulator of interferon genes (STING) signaling pathway has emerged as a key player against pathogens through detecting cytosolic DNA and prompting a robust immune response with the production of type I interferon (IFN-I) and other immune mediators. Beyond its involvement in host defense, the cGAS-STING signaling has been found to play a role in various physiological and pathological processes, including anti-tumor immunity, neurodegenerative diseases, and inflammatory diseases. However, it is still unclear whether the cGAS-STING pathway directly contributes to inflammaging and functional decline.

Recently, Gulen *et al*. [1] have confirmed the role of cGAS-STING axis in inflammaging and ageing-related neurodegeneration. Using a specific and well-tolerated STING inhibitor (H-151), they found that H-151 effectively inhibited the expression of IFN-1-stimulated genes (ISGs) and proinflammatory genes, without affecting other non-inflammatory features both in cellular (human WI-38 fibroblasts) and tissue (human adipose tissue) levels. Furthermore, STING inhibition by H-151 reduced inflammaging in the liver, kidney and brain, as well as significantly improved the physiological and cognitive functions in natural aged mice. Similar results were obtained in aged *Sting1* ^−/−^ mice [1], suggesting that STING is an important driving factor for ageing-related inflammation, and ageing-related functional decline.

**Figure 1:**
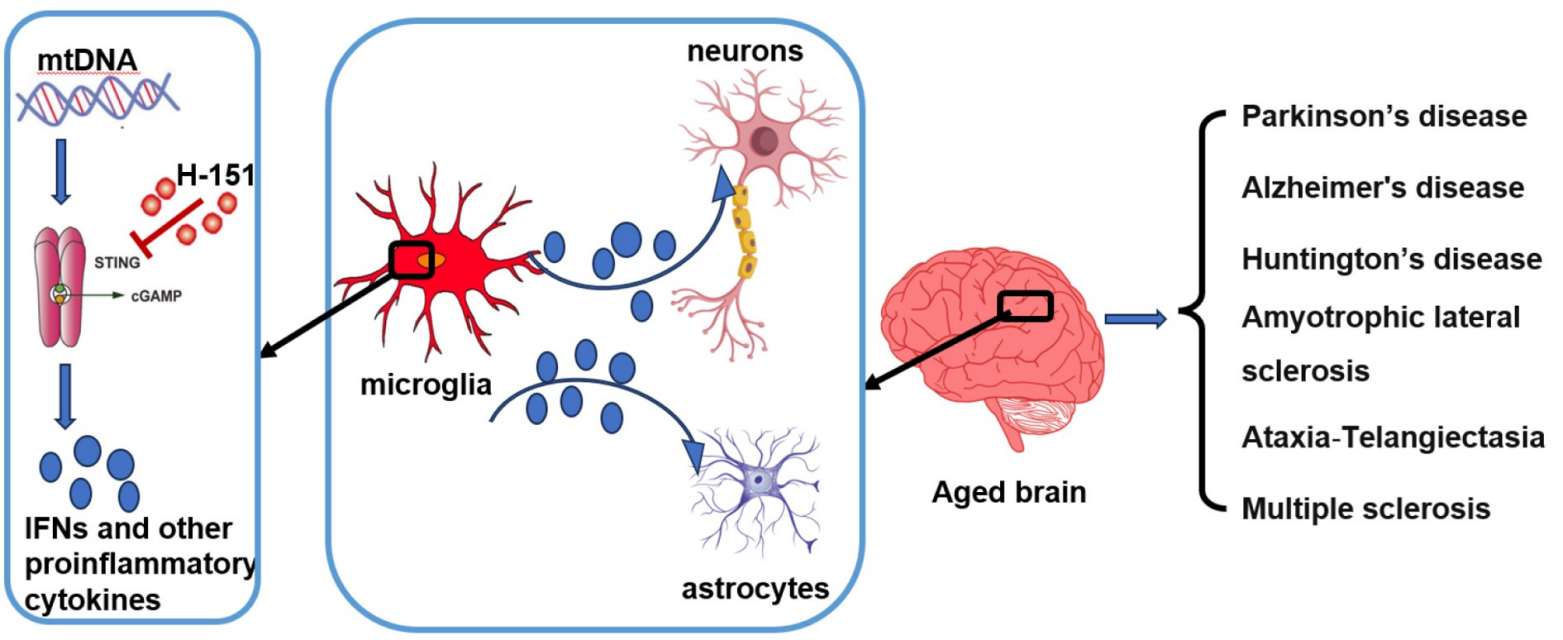
Figure 1 Role of mtDNA-cGAS-STING in neurodegenerative diseases Leakage of mitochondria DNA (mtDNA) in microglia stimulates the cGAS-STING pathway, resulting in production of IFNs and other cytokines, while H-151 inhibits STING and suppresses the signaling activation. Furthermore, proinflammatory cytokines impacts adjacent cells inflammation, neurotoxicity, causing various degenerative diseases.

It is widely acknowledged natural ageing is the pivotal risk factor for most neurodegenerative disorders. Microglias are the primary resident immune cells in the brain with high expression of STING and production of IFN-I and other proinflammatory cytokines. Gulen *et al*. [1] found that mitochondria DNA (mtDNA) triggered a cGAS-dependent neuroinflammation in aged microglial, establishing a significant association between mitochondrial dysfunction and inflammation, both of which are hallmarks of ageing and neurodegenerative disease. Using a mouse model expressing cGAS limited in microglia, they further revealed that besides production of IFN-1 in microglia, cGAS activation also promote ageing-related and neurodegenerative disease-related transcriptional states, leading to adjacent cells inflammation, neurotoxicity and impaired memory capacity. Meanwhile, STING inhibitor H-151 offers multiple benefits in promoting a healthy central nervous system (CNS), decreasing the levels of microglial cell, reducing the immunoreactivity of astrocytes, preventing the loss of neurons in the hippocampal CA1 region, and enhancing synaptic activity [1] ( Figure 1). In accordance with this finding, Szego *et al*. [2] also demonstrated that the density of dopaminergic axon terminals (fibers) and the concentration of dopamine in the striatum was lower in *STING* knock-in (ki) mice than in STING wild-type (WT) mice, while the concentration of dopamine metabolites was higher in *STING* ki mice, suggesting STING contributes to the loss of dopaminergic neurons in Parkinson’s disease (PD). It should be noted that besides mtDNA, micronuclei fragments could also trigger cGAS pathway in striatal neurons derived from human Huntington’s disease (HD) embryonic stem cells [3].

This project indicated that aberrant mtDNA-cGAS-STING is the common pathway triggering a neuroinflammatory microenvironment, which is crucial for the progress of a wide range of neurodegenerative diseases, such as PD, HD, Alzheimer’s disease (AD), as well as amyotrophic lateral sclerosis (ALS). For instance, activation of innate immune cGAS-STING pathway exacerbates amyloid-β plaques deposition, contributing to AD pathogenesis in 5xFAD mice [4]. Meanwhile, tau activates microglial cGAS-STING pathway and reduces neuronal myocyte enhancer factor 2c (MEF2C)-mediated cognitive resilience in AD [5]. Mutations in PINK1 and Parkin contribute to early-onset of PD. Borsche *et al*. [6] demonstrated that Parkin and PINK1 deficiency leads to impaired mitophagy and subsequent mtDNA release, thereby activating mtDNA-cGAS-STING pathway. Similarly, inappropriate activation of cGAS by self-DNA promotes autosomal recessive neurodegenerative disease such as Aicardi-Goutières syndrome (AGS) [7]. However, due to the distinctive molecular mechanisms underlying various neurodegenerative diseases, consequently, developing therapeutics targeting divergent upstream triggers or downstream effectors of cGAS-STING pathway is of critical importance [8]. For instance, a decreased nicotinamide adenine dinucleotide (NAD ^+^) level with compromised cellular bioenergetics and DNA repair contributes to the pathogenesis of AD, while supplementation with nicotinamide riboside (NR) normalizes NAD ^+^ concentration, subsequently reduces cytosolic DNA and the cGAS-STING signaling, and thereby inhibiting IFN-1-related neuroinflammation, indicating a new direction for the treatment of AD [9]. Similarly, in HD, mitochondrial oxidative stress with higher mtDNA release overactivates the cGAS, resulting in inflammation in brain and primary cerebro-cortical neurons, which is alleviated by melatonin, an endogenous free radical scavenger, working by maintaining mitochondrial homeostasis [10]. Of note, blocking gap junctions with carbenoxolone (CBX) can inhibit the transfer of cGAMP, reduce the activation of cGAS/STING in neighboring cells and interfere with the spread of inflammation in CNS [11]. Collectively, inhibiting the cGAS-STING pathway and its related signaling molecules presents a promising therapeutic approach for the treatment of neurodegenerative diseases ( Table 1).

**Table:** **Table 1** Therapeutic approaches targeting cGAS-STING pathway for neurodegenerative diseases

Disease	Intervention	Mechanism	Result	Reference
AD	NR	Stimulate mitophagy, and reduce mtDNA release and cGAS activation	NR supplementation improved cognitive and synaptic functions in APP/PS1 mutant mice.	[9]
HD	Melatonin	Decrease mitochondrial oxidative stress, reduce cytosolic DNA and decrease inflammation	Melatonin inhibited mtDNA-induced neuroinflammation in aralkylamine N-acetyltransferase knockout mice.	[10]
PD	Mdivi-1	Decrease mitochondrial fission, and reduce mtDNA leakage and cGAS activation	Mdivi-1 treatment inhibited DRP1 and maintained mitochondria membrane potential in Lrrk2 knockout macrophages.	[12]
AT	H-151	Inhibit STING	H-151 efficiently inhibited SASP expression, ameliorated astrocyte senescence, and alleviated A-T brain organoid neuropathology.	[13]
MS	Ganciclovir	Inhibit microglial reactivity and neuroinflammation	Ganciclovir (GCV) inhibited inflammation in MS mouse model dependent on STING-mediated IFN-1 response.	[14]
ALS	H-151	Inhibit STING, and decrease IFN-1 and NF-κB expressions	H-151 prevented neurons loss, ameliorating disease progression in ALS mouse model.	[15]

AD: Alzheimer’s disease; HD: Huntington’s disease; PD: Parkinson’s disease; AT: Ataxia-Telangiectasia; MS: multiple sclerosis; ALS: amyotrophic lateral sclerosis.

Importantly, there are still several issues need to be addressed before considering STING-based therapeutics in neurodegenerative disease. Besides initiating immune responses, there is an complicated crosstalk between cGAS-STING pathway and various modes of cell death during microbial infections, which is reviewed in a previous study [16]. STING activation directs autophagy and subsequent degradation, preventing sustained STING activation [16]. Is it possible that STING-mediated autophagy can be an effective approach for decreasing inflammation? Normal CNS function requires the involvement of the immune system, however, excessive immune activation can be detrimental [8]. Thus, cGAS-STING signaling is a double-edged sword, as it can be either beneficial or detrimental to nearly every organ system depending on different contexts of its activation (self-DNA or foreign DNA), cell-type, the degree of inflammatory responses, and the duration of activation. Taken together, a better understanding of the regulatory mechanism will ultimately pave the way for the use of STING-based treatments.
